# Sleep: the neglected life factor in adults with intellectual disabilities

**DOI:** 10.1192/bjb.2021.122

**Published:** 2023-06

**Authors:** Laura Korb, David O'Regan, Jane Conley, Emma Dillon, Rachel Briggs, Ken Courtenay, Bhathika Perera

**Affiliations:** 1 Barnet, Enfield and Haringey Mental Health NHS Trust, UK; 2 Guys and St Thomas’ NHS Foundation Trust, UK; 3 The Tavistock and Portman Trust, UK; 4 University of Reading, UK

**Keywords:** Intellectual disabilities, sleep disorders, insomnia, neurodevelopmental disorder, learning disability

## Abstract

Sleep is vital for our physical and mental health. Studies have shown that there is a high prevalence of sleep disorders and sleep difficulties amongst adults with intellectual disabilities. Despite this, sleep is often overlooked or its disorders are considered to be difficult to treat in adults with intellectual disabilities. There is a significant amount of research and guidance on management of sleep disorders in the general population. However, the evidence base for sleep disorders in adults with intellectual disabilities is limited. In this review paper, we look at the current evidence base for sleep disorders in adults with an intellectual disability, discuss collaborative working between intellectual disabilities psychiatrists and sleep medicine specialists to manage sleep disorders, and provide recommendations for future directions.

Sleep disorders, including sleep-disordered breathing and insomnia, are more common in adults with intellectual disabilities when compared with the general population,^1^ with one systematic review finding that 32% of individuals with intellectual disabilities experienced multiple sleep problems.^2^ Adults with intellectual disabilities experience more fragmented sleep compared with the general population,^2^ and those with more severe intellectual disabilities are at higher risk of sleep disturbance.^3,4^ Although adults with intellectual disabilities are a heterogenous group, specific sleep disorders can cluster around genetic syndromes or disorders as well as comorbid neurodevelopmental disorders.^1^

Aside from their well-described physical and mental health consequences, sleep disorders may also contribute to challenging behaviours in adults with intellectual disabilities and increase carer burden.^5–7^ This combination of disturbed nocturnal sleep, increased challenging behaviours and carer distress frequently result in higher social care and healthcare utilisation.^8^

Despite the higher prevalence of sleep disorders and their associated sequelae in adults with intellectual disabilities, there remains a paucity of research and guidance regarding the optimal approaches towards their assessment and management.^9^ In this narrative review, we explore the current evidence base for assessing, diagnosing and treating sleep disorders in adults with an intellectual disability and share our experience of collaborative working between intellectual disabilities psychiatrists and sleep medicine specialists.

## Sleep disorders in people with intellectual disability

Sleep problems are common in people with intellectual disabilities, with the prevalence of sleep problems in children ranging from 24% to 86%.^2,10–13^ The estimated prevalence of sleep disorders in adults with intellectual disabilities ranges from 8.5% to 34.1%, with 9.2% experiencing significant sleep problems.^2^ Van de Wouw et al^2^ found that 72% of 551 older adults with an intellectual disability had sleep difficulties.

Mental and physical health disorders and their treatment in people with intellectual disabilities represents an under-researched area. Diagnostic and management strategies are commonly adopted from studies undertaken in people without intellectual disabilities. Sleep disorders in adults with intellectual disabilities follow the same pattern. Richdale et al^10^ described the disappointing lack of research specifically concerning the aetiology and impact of sleep difficulties and the corresponding interventions in those with developmental disorders. Understanding the types of sleep problems that adults with intellectual disabilities experience and the many factors that influence their sleep can inform the assessment and management of sleep problems in adults with intellectual disabilities.^14^

**Fig. 1:**
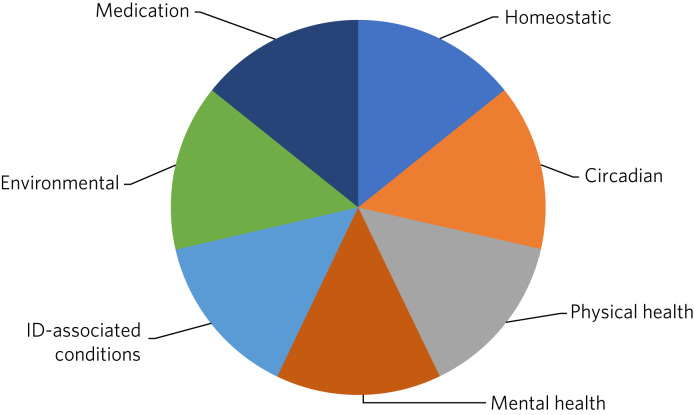
Factors affecting sleep in people with intellectual disabilities (ID).

**Fig. 2:**
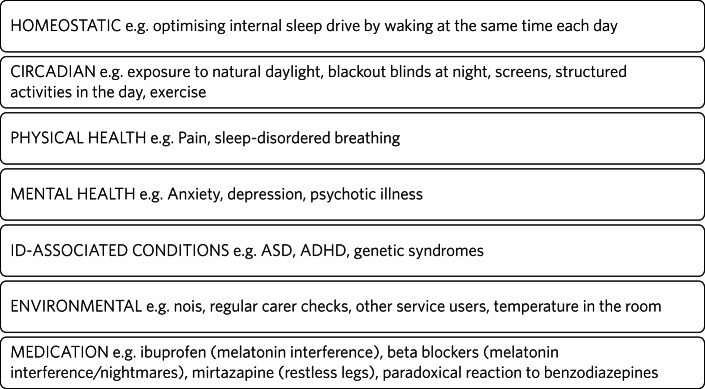
Examples of factors affecting sleep in people with intellectual disabilities (ID).

There are several potential explanations of why adults with intellectual disabilities are more likely to experience sleep problems. A systematic review of the published literature on sleep disorders in adults with intellectual disabilities by Van de Wouw et al^2^ reported associations between sleep and several factors, including challenging behaviour, psychotropic medication, mental health conditions and respiratory diseases. To provide person-centred and individualised care, it is important to understand and consider the biological, psychological and social factors contributing to the increased prevalence of sleep problems in adults with intellectual disabilities (Figs. 1 and 2). We have explored several important contributing factors that should be considered when assessing sleep difficulties in adults with intellectual disabilities.

### Neurodevelopmental conditions

Autism spectrum disorder (ASD) is a common comorbidity in people with intellectual disabilities, with an estimated prevalence of up to 39% in adults with moderate to profound intellectual disabilities.^15^ Sleep problems that persist across a lifetime commonly occur in individuals with ASD.^16^ Ballester et al^17^ compared circadian rhythms and sleep patterns in adults with ASD and intellectual disabilities with those of typically developing adults. Their findings indicated that adults with ASD and intellectual disabilities have prolonged sleep onset latency, increased frequency and length of night awakenings, and low sleep efficiency in comparison with adults without a neurodevelopmental disorder. A review by Cohen et al^18^ discussed the multi-factorial aetiology for sleep problems in those with ASD, including evidence of biological abnormalities in the timing of melatonin secretion and sleep disruption secondary to co-occurring medical and psychiatric conditions.

In addition, individuals with intellectual disabilities have a higher prevalence of attention-deficit hyperactivity disorder (ADHD) than those without intellectual disabilities, with reported rates of up to 19.6%.^19^ Sleep problems are a common issue affecting people with ADHD, reported in up to 50% of people with this condition.^20^ Many reasons have been proposed as to why people with ADHD experience sleep difficulties. Hvolby et al^21^ postulated that the relationship between sleep and ADHD is multi-faceted and that disturbed sleep could be considered to be an intrinsic feature of ADHD, further complicated by the effects of psychostimulant medication. Despite this known association, sleep problems relating to ADHD may be labelled as ‘challenging behaviour’ in individuals with comorbid intellectual disabilities.^22,23^

### Genetic conditions

Recently, our understanding of the underlying genetic factors in the development of intellectual disabilities has improved.^24^ For example, the characteristic features of Down's syndrome include hypotonia, obesity and craniofacial abnormalities, all of which increase the risk of sleep-disordered breathing such as obstructive sleep apnoea (OSA).^25^ Similarly, patients with *cri du chat* syndrome are at increased risk of developing OSA.^26^ Patients with Smith–Magenis syndrome are at increased risk of circadian sleep–wake disorders, classically presenting with an inverted sleep–wake cycle, which is thought to be caused by an aberrant melatonin pathway.^27^

### Psychological and environmental factors

Sleep disturbance can often be the first sign of a deterioration in the mental state of people with mental illness, and poor sleep is a core symptom of many affective and psychotic disorders.^28^ The high prevalence of sleep disorders in people with intellectual disabilities can be understood in the context of the higher prevalence of mental illnesses in people with intellectual disabilities, compared with those without intellectual disabilities.^29^

The role of the environment in the genesis of sleep disorders is an important factor to consider when identifying the aetiology of sleep problems in a person with intellectual disabilities. Kerr and Wilkinson^30^ identified that staffed residential homes may not be ideal for sleeping because staff may check on residents during the night, resulting in increased noise and lighting that disturbs sleep. Other environmental factors to consider for a person living in a supported home include the likelihood of living with other people with sleep difficulties who may make loud noises overnight, lack of access to outdoor activities that expose the individual to natural light, and the reliance on support staff to consider simple measures such as black-out blinds.

## Diagnosing sleep disorders

Although there are guidelines on the diagnosis and management of sleep disorders in the non-intellectual-disabilities population,^31,32^ intellectual-disabilities-specific guidelines are lacking. More often than not in adults with intellectual disabilities, subjective sleep information is provided by carers, who may have differing opinions on the level of sleep disturbance or may even simply accept sleep disturbance as part of the person's underlying condition.^33^ Consequently, sleep disorders are likely to be brought to clinician's attention when they lead to nocturnal and daytime dysfunction, including behavioural disturbance, rather than because of their impact on the person's subjective opinion of their quality of life.^34^ Questionnaires such as the Insomnia Severity Index^35^ can be used to assess severity and monitor response to treatment, but they are rarely validated in people with intellectual disabilities and rely more on carers’ reports.

Guidelines aimed at the general population emphasise the importance of looking for comorbid medical conditions when assessing sleep disorders.^32^ This is arguably even more important in individuals with intellectual disabilities, who are more likely to have a physical health condition such as OSA or epilepsy which affects their sleep.^36–38^ For example, owing to the high prevalence of OSA in individuals with Down's syndrome, it is recommended that everyone with Down's syndrome is screened for this condition.^36^

Sleep diaries completed by carers and/or actigraphy, ideally undertaken for a minimum of 2 weeks, can be used when sleep–wake timings (including napping) are inconsistent or unreliable.^39^ Home or in-patient sleep investigations (for example, pulse oximetry or the gold-standard, polysomnography) can be used to investigate physical sleep disorders such as OSA and nocturnal epilepsy in people with intellectual disabilities.^40^ While these investigations should always be offered when clinically appropriate, a pragmatic trial of treatment may sometimes be required when sleep investigations are not tolerated by the individual.

**Fig. 3:**
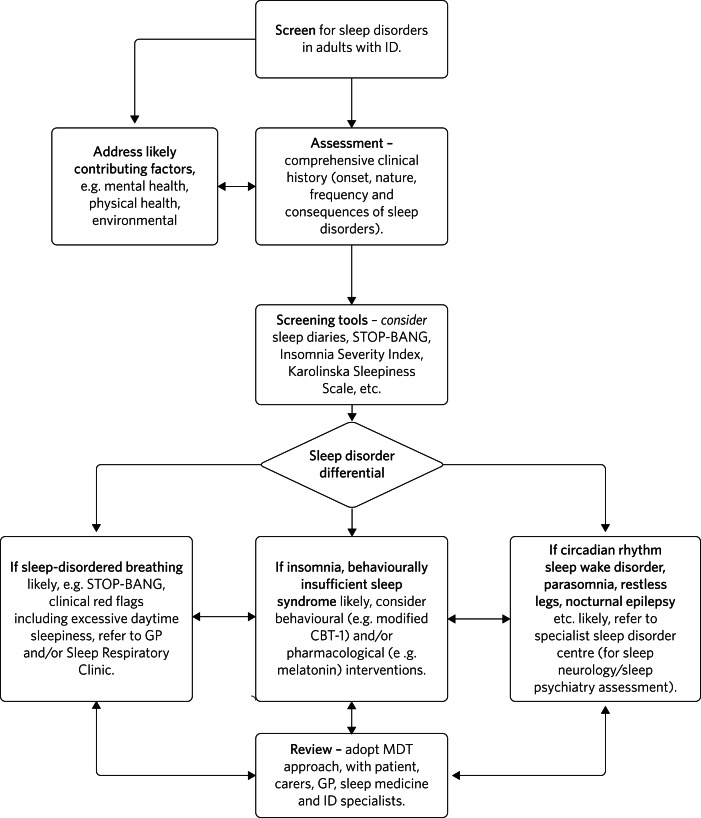
Flowchart for screening, assessing and managing sleep disorders in adults with intellectual disabilities (ID). GP, general practitioner; MDT, multidisciplinary team.

In Fig. 3, we outline a suggested approach for the assessment and management of sleep disorders in adults with intellectual disabilities, based on our collective clinical experience.

## Management strategies for sleep disorders in people with intellectual disabilities

The management of sleep disorders is complicated by the heterogeneity of the underlying causes of intellectual disabilities, along with the nature of associated comorbidities. Ideally, each of the factors outlined in Fig. 1 should be addressed in order to optimise sleep, as illustrated by the case vignette (Box 1). Where several factors are driving sleep disturbance, solely focusing on one factor is unlikely to lead to satisfactory resolution. Whereas some factors can be readily addressed by intellectual disabilities psychiatry, other factors will require support from sleep medicine specialists and/or primary care. Again, there is a paucity of research in this area.^9^

For adults with Down's syndrome and OSA, a study provides evidence that the use of continuous positive airway pressure therapy can lead to significant improvements in subjective sleepiness, behaviour and cognitive function.^41^ However, as rightly acknowledged in this study, treatment may be challenging to access and, as previously noted, there may be some patients with intellectual disabilities that struggle to tolerate these investigations and interventions. Such challenges may be overcome by sleep physicians and psychiatrists working collaboratively. For example, our sleep clinic is helping to train intellectual disabilities mental health nurses in exposure therapy to help patients acclimatise to positive airway pressure masks.

Multicomponent cognitive–behavioural therapy for insomnia (CBT-I) is the first-line treatment for chronic insomnia in the general population, and there are aspects of this that can be useful for individuals with intellectual disabilities^31,32^ (Table 1). Education on sleep hygiene can be effective in helping the person and carer understand the effects of lifestyle and environment on sleep, for example, caffeine intake, exercise and regular sleep routine, along with lighting, noise and temperature.^34^ Even modest adaptations to daily routine and the sleeping environment can benefit adults with intellectual disabilities, who are more likely to lack adequate daily exercise and regular exposure to natural light compared with the general population.^34^ Such advice should be tailored to the needs of the person, for example, interventions to reduce environmental noise for autistic people who are likely to be sensitive to sound.^34^

**Table 1:** Modified CBT-I for adults with intellectual disabilities

Technique	Method	General advice
Anchoring the day	Setting a fixed rising time that is maintained 7 days a week, no matter how tired the individual is or how little they have slept. *This technique aims to optimise the homeostatic sleep drive.*	Easier to achieve with the aid of carers and if there is something to get up for, i.e. structured daytime activity. We recommend setting an alarm so the anchor time is kept constant.
Natural light exposure	Ideally, within 2 h of rising, the individual gains access to natural light (even if it seems dull outside). A minimum of 20 min is recommended. *This technique aims to optimise the circadian rhythm.*	Easier to achieve if there is structured daytime activity with regular meal times (which also feeds into the circadian clock). If it is possible to walk to daytime activities (e.g. college, day centre, etc.) or schedule exercise for this time, it can become part of the individual's usual routine.
Stimulus Control	Ideally, the bed and bedroom should be kept for sleep, intimacy and getting dressed *only*. All other activities should be kept outside the bed/bedroom. *This technique is based on classical conditioning, and aims to re-establish that the bed/bedroom is a place for sleep as opposed to a place for wakeful activities.*	If the individual has access to only one room, encourage them not to sit on the bed or use it for activities other than sleep. The bedroom can be made to look different in the day as opposed to the night (e.g. using a different bed cover during the day, or placing a plant in the room during the day and taking it away at night). This will help the mind to know when the room is in day or night mode.
Buffer zone	This is a period, usually of at least 90 min before bed, where the body and mind are moved into a state of relaxation ready for sleep. We advise beginning the buffer zone with a warm bath and afterwards keeping rooms dimly lit, and letting the individual engage in relaxing (i.e. non-stimulating) activities before it is time for bed.	If the individual does not have access to a bath, then trialling a shower can also be helpful. A risk assessment must be carried out prior to recommending bathing, e.g. it may be unsuitable for some individuals with epilepsy.
Sleep rescheduling	In this technique, the total sleep time is closely matched to the total time in bed (often by keeping sleep diaries and/or actigraphy). This will increase the internal sleep drive (in conjunction with anchoring the day) and reduce hyperarousal. This technique has been used on its own and as part of multimodal CBT-I to good effect in adults with intellectual disabilities.^34^	This technique may require careful carer education, as often adults with intellectual disabilities have bedtimes that are too early forced upon them. It should be used with caution in adults with intellectual disabilities who have comorbidities which can be made worse by temporary sleep restriction/loss, e.g. epilepsy, migraine, bipolar affective disorder.^42^
Miscellaneous	Engagement in regular exercise (ideally getting out of breath if safe to do so). Structured daytime activity. Regular meal times. Discourage eating during the night if the individual cannot sleep or giving extra attention at this time point.	All of the techniques in this table require patience and persistence in order to be effective. Initially sleep may worsen, as the usual routine is being changed, which can be anxiety-provoking. Trialling one technique at a time may mitigate this potential difficulty.

The evidence for the pharmacological management of sleep disorders in adults with intellectual disabilities is not well established. The medication which has received the most attention is melatonin, probably because of its favourable side-effect profile, and some studies have shown it to be effective.^34^ A meta-analysis by Braam et al^43^ concluded that in individuals with intellectual disabilities, the use of melatonin decreases sleep latency and the number of wakes per night, and increases the total sleep time. At present, the pharmacological management of non-insomnia disorders tend to follow the same pathways as those for the general population.^31^

Box 1 Case Vignette

Patient X is a 26 year-old man with a moderate intellectual disabilities, ADHD and Smith–Magenis syndrome. He presented with a disrupted sleep pattern (akin to day/night reversal), aggression and hyperactivity. Attempts were made to optimise his environment by limiting evening screen time, using black-out blinds and maintaining a regular exercise regime. Medication had been trialled, including acebutolol (to block daytime melatonin production) in the morning combined with circadin (i.e. modified-release melatonin) at night. Unfortunately, owing to aggression towards staff and other service users, he was excluded from his day centre. His carers were struggling to engage him during the day and were unable to manage his poor sleep pattern, which included habitual snacking at night. He was referred to sleep psychiatry at a sleep disorder clinic. Non-invasive investigations were performed to exclude sleep-disordered breathing, acebutolol was stopped and he was successfully started on agomelatine. Techniques to support an improved sleep pattern were advised, including: delayed bed time, morning light exposure and structured daytime activity (i.e. a new day centre and exercise), and staff were encouraged not to engage with him at night if he awoke, which helped to eliminate night-time snacking. His sleep–wake pattern improved (i.e. sleep maintenance, total sleep time and sleep efficiency) as did his daytime function (i.e. mood, hyperactivity and behaviour).

## Conclusion

Sleep disorders are common in adults with intellectual disabilities, where they adversely affect mental and physical well-being as well as daytime functioning. Sleep disturbances are often multifactorial and, despite their negative sequelae, they are often considered to be secondary rather than primary diagnoses.

When assessing and managing sleep disorders in adults with intellectual disabilities, clinicians often have to rely on information that has been gathered from carers rather than from the patient themselves, which may lead to a biased assessment. Similarly, the success of intervention delivery will in most cases be dependent on the knowledge, ability and willingness of caregivers.

There is a lack of robust evidence in the field concerning both non-pharmacological and pharmacological strategies for managing sleep disorders in adults with intellectual disabilities. The proposed flowchart shown in Fig. 3 for screening, assessing and managing sleep disorders may provide clinicians with a structured approach. Further research on sleep disorders in adults with intellectual disabilities is required. A validated tool to screen for and assess sleep disorders in adults with intellectual disabilities would be invaluable, particularly for people who are unable to communicate their difficulties.

## Key points

- Sleep disorders are common in adults with intellectual disabilities.
- Sleep disorders can adversely affect the overall health and quality of life of adults with intellectual disabilities.
- Sleep disorders are often treated as a part of a mental disorder rather than specifically screened for and managed.
- More research into the assessment and management of sleep disorders in adults with intellectual disabilities is required.

## About the authors

**Laura Korb** is a Consultant Psychiatrist in Intellectual Disabilities at Barnet, Enfield and Haringey Mental Health NHS Trust, UK; **David O'Regan** is a Consultant in Psychiatry and Sleep Medicine at Guys and St Thomas’ NHS Foundation Trust, UK; **Jane Conley** is a Core Trainee in Psychiatry at Barnet, Enfield and Haringey Mental Health NHS Trust, UK; **Emma Dillon** is a Trainee IAPT Therapist for Autism and Learning Disabilities at the Tavistock and Portman Trust, UK; **Rachel Briggs** is a trainee children's well-being practitioner at the AnDY research clinic, University of Reading, UK; **Ken Courtenay** is a Consultant Psychiatrist in Intellectual Disabilities at Barnet, Enfield and Haringey Mental Health NHS Trust, UK, and the Chair of the Faculty of Intellectual Disabilities, Royal College of Psychiatrists, UK; **Bhathika Perera** is a Consultant Psychiatrist in Intellectual Disabilities and ADHD at Barnet, Enfield and Haringey Mental Health NHS Trust, UK.
